# Homologous recombination deficiency (HRD) is associated with better prognosis and possibly causes a non‐inflamed tumour microenvironment in nasopharyngeal carcinoma

**DOI:** 10.1002/2056-4538.12391

**Published:** 2024-08-05

**Authors:** Xinyi Zhou, Haoxuan Ying, Yujie Sun, Wenda Zhang, Peng Luo, Shuhan Zhu, Jian Zhang

**Affiliations:** ^1^ Department of Oncology Zhujiang Hospital, Southern Medical University Guangzhou PR China

**Keywords:** nasopharyngeal carcinoma, homologous recombination deficiency score, prognostic value, non‐inflamed tumour microenvironment, platinum compounds and PARP inhibitors, precision treatment, si‐BRCA1, sensitivity to chemoradiotherapy

## Abstract

Homologous recombination deficiency (HRD) score is a reliable indicator of genomic instability. The significance of HRD in nasopharyngeal carcinoma (NPC), particularly its influence on prognosis and the immune microenvironment, has yet to be adequately explored. Understanding HRD status comprehensively can offer valuable insights for guiding precision treatment. We utilised three cohorts to investigate HRD status in NPC: the Zhujiang cohort from local collection and the Hong Kong (SRA288429) and Singapore (SRP035573) cohorts from public datasets. The GATK (genome analysis toolkit) best practice process was employed to investigate germline and somatic *BRCA1/2* mutations and various bioinformatics tools and algorithms to examine the association between HRD status and clinical molecular characteristics. We found that individuals with a negative HRD status (no‐HRD) exhibited a higher risk of recurrence [hazard ratio (HR), 1.43; 95% confidence interval (CI), 2.03–333.76; *p* = 0.012] in the Zhujiang cohort, whereas, in the Singapore cohort, they experienced a higher risk of mortality (HR, 26.04; 95% CI, 1.43–34.21; *p* = 0.016) compared with those in the HRD group. *In vitro* experiments demonstrated that NPC cells with BRCA1 knockdown exhibit heightened sensitivity to chemoradiotherapy. Furthermore, the HRD group showed significantly higher tumour mutational burden and tumour neoantigen burden levels than the no‐HRD group. Immune infiltration analysis indicated that HRD tissues tend to have a non‐inflamed tumour microenvironment. In conclusion, patients with HRD exhibit a comparatively favourable prognosis in NPC, possibly associated with a non‐inflammatory immune microenvironment. These findings have positive implications for treatment stratification, enabling the selection of more precise and effective therapeutic approaches and aiding in the prediction of treatment response and prognosis to a certain extent.

## Introduction

Nasopharyngeal carcinoma (NPC) is an epithelial tumour that exhibits a distinctive geographical distribution, primarily prevalent in Southern China and Southeast Asian regions [1]. Numerous research endeavours are currently focused on investigating cancer genomics through whole exome sequencing (WES), including in NPC. Homologous recombination deficiency (HRD) testing has gained significant traction in diagnosing and treating various cancers, such as breast, ovarian, prostate, and pancreatic cancers [2]. Studies have shown that HRD can guide the application of platinum and PARP inhibitors (PARPi) and predict the prognosis and treatment response to some extent in breast, ovarian, prostate, and pancreatic cancers [3, 4, 5, 6, 7, 8]. However, limited studies have been conducted to explore the HRD status of NPC and its consequent clinical implications.

Based on a comprehensive study utilising data from the cancer genome atlas (TCGA) and encompassing 8,847 patients across 33 different types of cancers, an HRD score threshold of 42 was employed to discern the presence of HRD in the samples. Notably, 1,552 samples (17.5%) were identified as HRD‐positive. Specifically, the prevalence of HRD‐positive cases in head and neck squamous cell carcinoma (HNSCC) was determined to be 23%, positioning it as the 10th most prevalent among the 33 cancer types examined, indicating that HRD status is relatively essential in HNSCC [2].

The primary therapeutic approach for NPC involves the utilisation of radiotherapy in conjunction with platinum‐based chemotherapy, encompassing induction chemotherapy, concurrent chemoradiotherapy, and adjuvant chemotherapy [1]. A prominent mechanism underlying the efficacy of this treatment modality is the induction of DNA damage. The homologous recombination repair (HRR) pathway is a pivotal component within the broader DNA damage repair pathway, a fundamental metabolic process across all biological organisms [9]. Generally speaking, individuals with HRD are more susceptible to DNA damage treatments involving platinum and PARPi [2, 4, 10]. From this perspective, it is necessary to investigate the status of HRD in NPC to facilitate a more rational utilisation of drugs that specifically target DNA damage, such as platinum and PARPi.

In this study, patients were primarily grouped based on their HRD status to investigate the variations in age at first diagnosis, tumour mutational burden (TMB), tumour neoantigen burden (TNB), microsatellite instability (MSI) score, and chromosomal ploidy among patients with different HRD statuses. Additionally, the study aimed to determine if HRD status could serve as an independent prognostic factor for NPC patients and to preliminarily explore the impact of HRD on the tumour immune microenvironment of NPC. Finally, we knocked down BRCA1 expression in NPC cells to induce a relative deficiency in HRR function to investigate alterations in cell sensitivity towards chemoradiotherapy.

## Materials and methods

### Patient cohorts and clinical information

A total of 58 NPC samples were prospectively collected at Zhujiang Hospital. Among these, 19 primary samples were mainly obtained from biopsy tissues, whereas 38 were recurrent samples primarily obtained from surgical resection. Additionally, one sample could not be definitively classified as either primary or relapsed due to an error during collection. The local Zhujiang cohort included 41 tumour tissue transcriptome (mRNA) sequencing data from 58 paired samples. The Zhujiang cohort serves as a local discovery dataset. The Singapore cohort [11] obtained from the Sequence Read Archive (SRA) database (accession no. SRP035573) (including 56 primary NPC patients) and the Hong Kong cohort [12] obtained from the SRA database (accession no. SRA288429) (including 50 primary NPC patients) were used as validation datasets. All samples from three cohorts consisted of WES data derived from tumour tissues and corresponding peripheral blood. In the Zhujiang cohort, all patients provided written informed consent for genome analysis, and the research project received approval from the Ethics Committee of Zhujiang Hospital, with a registration number of ChiCTR2000028838 at the China Clinical Trial Center (https://www.chictr.org.cn/index.html). Clinical data were retrospectively extracted from electronic medical records.

### WES and genomic data processing

For WES data from the local Zhujiang cohort, the SureSelect Target Enrichment Kit V6 Human Whole Exome Capture Kit (Agilent, Santa Clara, CA, USA) was utilised for library construction. Following quality control, the library was subjected to sequencing on the NovaSeq6000 instruments (Illumina, San Diego, CA, USA) and NovaSeq S4 reagent kit (Illumina) according to the manufacturer's instructions with 150‐bp paired‐end reads and a mean coverage of 100×. HaploX Biotechnology Co., Ltd. (Shenzhen, Guangdong, PR China) completed the sequencing workflow of the Zhujiang cohort.

The raw sequencing data were aligned to the reference genome GRCh38 using the Burrows‐Wheeler‐Alignment Tool (BWA0.7.17‐r1188) to generate the sam file [13]. Subsequently, the sam file was sorted, and duplicates were removed using Picard (2.18.29‐SNAPSHOT) to obtain bam files. Base quality score recalibration (BQSR) was conducted using gatk BQSR (gatk BaseRecalibrator), and the identification of germline and somatic mutations followed the gatk (gatk‐4.2.3.0) best practice process. The HaplotypeCaller tool was employed to detect germline mutations and generate a joint callset in VCF format. Subsequently, gatk VQSR or gatk VariantRecalibrator (variant quality score recalibration) was utilised to filter variants based on quality, following the recommended parameters provided by the official website. Somatic mutations were identified using Mutect2, and all obtained mutations underwent filtration using gatk GetPileupSummaries. The gatk CalculateContamination tool was employed to determine the proportion of cross‐contamination in each tumour sample [14].

The obtained final mutations were annotated using ensembl‐vep 104.3 [15] and subsequently imported into Rstudio for further statistical analysis utilising the R package maftools [16]. The average sequencing depths for the Zhujiang, Singapore, and Hong Kong cohorts were 100×, 86×, and 70×, respectively. The public files employed for the analysis were downloaded from the GATK resource bundle, accessible through the Google Cloud bucket hosted at https://console.cloud.google.com/storage/browser/gatk‐best‐practices. All the steps of specific analysis tools and accessible source links can be found in supplementary material, Supplementary links.

### Calculation of HRD score

Tumour purity and ploidy were determined using Sequenza 3.0.0. Previous research has demonstrated a correlation between chromosome ploidy and tumour malignancy [17]. The tumour purity/cellularity and ploidy data for the three cohorts are provided as supplementary material, Table S1. We utilised the R package scarHRD in Rstudio to calculate the HRD score with the seqz files [18].

The calculation of HRD score is an established approach for assessing the state of HRD. This score incorporates the assessment of genomic instability through the integration of loss of heterozygosity (LOH), number of large‐scale transitions (LST), and telomeric allelic imbalance (TAI). The specific value of the HRD score is determined by detecting and calculating the intracellular single nucleotide polymorphism sites. Individually, LOH, LST, and TAI can each serve as predictors of genome stability. However, the HRD score, the sum of three indicators, provides a more comprehensive reflection of genomicinstability. Other relevant and detailed methodological sections can be found in the Supplementary materials and methods [19, 20, 21, 22, 23, 24, 25, 26, 27, 28, 29].

In this study, the evaluation criteria for HRD refer to the diagnostic criteria of MyChoice CDx, commonly used in clinical practice, and the detail is as follows: HRD positivity was determined when a pathogenic *BRCA1/2* mutation was detected or when the cumulative score of the three indicators reached or exceeded 42. This positive status was denoted as HRD or HRD‐positive. Conversely, HRD negativity was assigned when no pathogenic mutation was found or the cumulative score was below 42. This negative status was expressed as no‐HRD or HRD‐negative.

### Statistical and survival analysis

The experimental data in this study underwent statistical analysis using R version 4.0.3. The Shapiro–Wilk test was employed to assess the normality of the measures. In cases where the data exhibited normal distribution, the *F*‐test was conducted to verify the homogeneity of variance. If the data exhibited normal distribution and homogeneity of variance, a two‐tailed unpaired *t* test was employed as a parametric test. Conversely, if the data did not meet the normality or variance homogeneity criteria, the non‐parametric Wilcoxon rank test was utilised. The Chi‐square test or Fisher's exact test was employed, as appropriate, to compare clinical and molecular characteristics between the HRD and no‐HRD groups in the count data. The relapse‐free survival (RFS) of patients in the Zhujiang cohort was assessed from the initial treatment date until disease progression, relapse or death, primarily based on imaging data and determined by physicians and radiologists. Patients were censored at the last follow‐up date or on 28 February 2022. The Singapore cohort's survival data (overall survival [OS]) were obtained from the originally published documentation [11]. The Kaplan–Meier method was employed to estimate both OS and RFS, while subgroup comparisons were conducted using the log‐rank test.

Univariate and multivariate survival analyses were conducted using the Cox proportional hazards model with likelihood test. A multivariate model for RFS was established in the Zhujiang cohort, whereas a multivariate model for OS was established in the Singapore cohort. These models incorporated known risk factors, including age at diagnosis and stage. *p* < 0.05 was considered statistically significant, denoted by ‘*’. For others, *p* < 0.1, denoted as ‘.’, *p* < 0.01, denoted as ‘**’, *p* < 0.001, denoted as ‘***’, and *p* < 0.0001, denoted as ‘****’. The statistical methods used and statistical significance are reported in each chart.

## Results

### HRD status of NPC patients in the three cohorts

**Figure 1:**
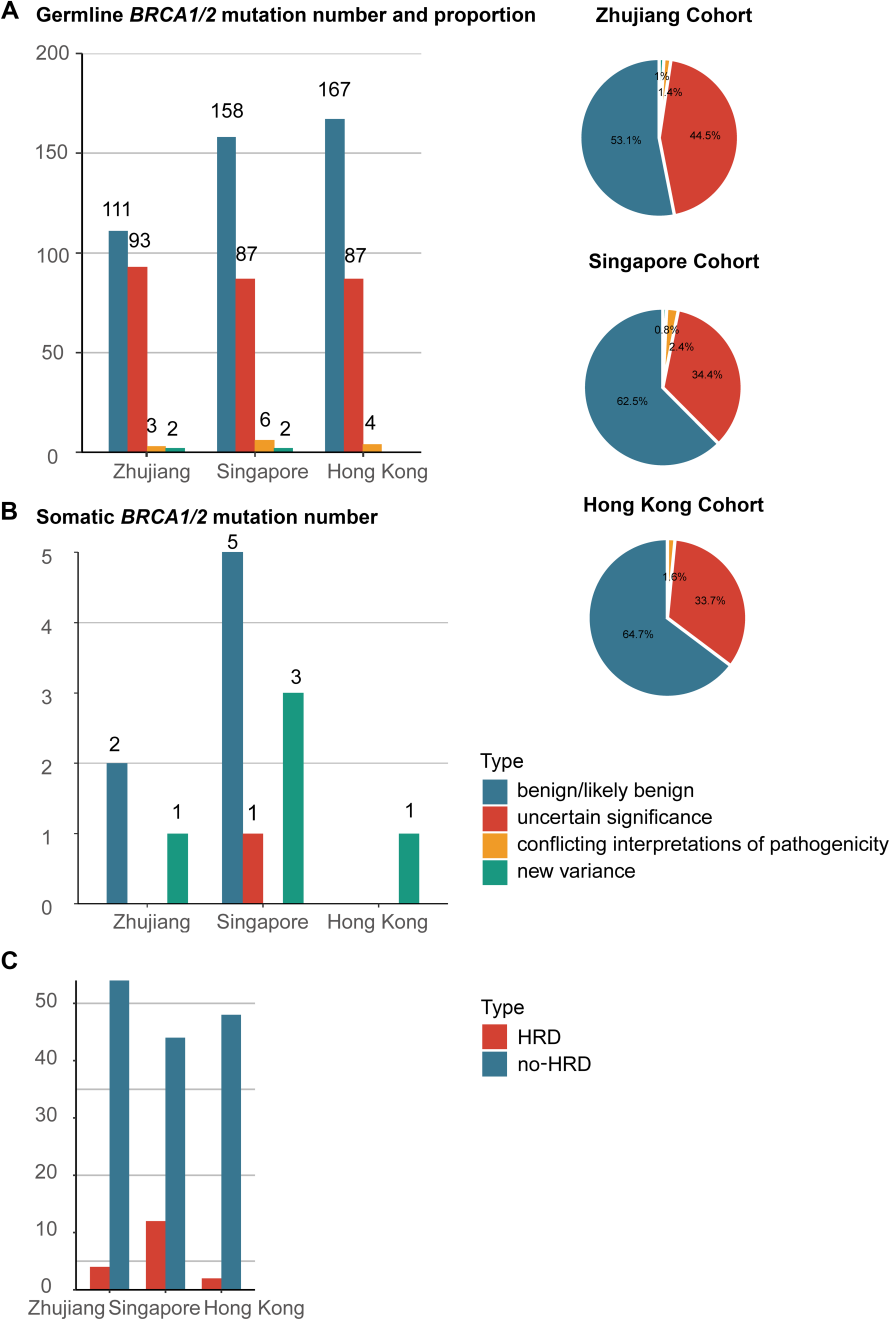
The quantity of *BRCA1/2* mutations and the prevalence of HRD tumours across the three cohorts. (A) Bar or pie charts illustrating the distribution of *BRCA1/2* germline mutations categorised by the clinical significance of mutations across the three cohorts. (B) Bar chart presenting the quantity of *BRCA1/2* somatic mutations categorised by the clinical significance of mutations in the three cohorts. (C) Bar chart displaying the number of patients with HRD versus those without HRD within the three cohorts. HRD, homologous recombination deficiency; no‐HRD, without homologous recombination deficiency.

The GATK best practice protocol was employed to identify *BRCA1/2* germline or somatic mutations within the three cohorts. Our analysis revealed a higher prevalence of *BRCA1/2* germline mutations than somatic mutations. The findings suggest a higher likelihood of germline *BRCA1/2* mutations rather than somatic mutations in NPC. Our analysis primarily focused on the clinical significance of five distinct categories of *BRCA1/2* mutations, namely benign/likely benign, uncertain significance, conflicting interpretations of pathogenicity, pathogenic/likely pathogenic, and new variants. The precise numerical values and corresponding proportions of mutations are depicted in Figure 1A–C. Remarkably, none of these mutations was deemed pathogenic or likely pathogenic based on existing databases. We reviewed the relevant literature in a pan‐cancer study that counted the prevalence of homologous recombinant DNA damage repair (HR‐DDR) defects across 21 cancer lineage. The overall frequency of HR‐DDR mutations detected was 17.4%; *ARID1A* was the most commonly mutated gene (7.2%), followed by *BRCA2* (3.0%), *BRCA1* (2.8%), *ATM* (1.3%), *ATRX* (1.3%), and *CHEK2* (1.3%) [30], indicating that more BRCAness genes like *ARID1A* within the HRR pathway are crucial for maintaining genomic stability in NPC.

Waterfall plots were employed to visually represent the germline (supplementary material, Figure S1) and somatic (supplementary material, Figure S2) mutations of 17 prominent genes associated with the HRR pathway across three cohorts [7]. These plots encompassed all mutations without emphasising their clinical significance. By combining the outcomes of *BRCA1/2* mutations and the HRD score, it was determined that 4 of 58 individuals (6.9%) in the Zhujiang cohort, 12 of 56 individuals (21.43%) in the Singapore cohort, and 2 of 50 individuals (4%) in the Hong Kong cohort were HRD‐positive (Figure 1D).

### Differences in clinical and molecular characteristics between different HRD statuses in the three cohorts

TMB has been identified as a valuable biomarker for utilising immune checkpoint inhibitors (ICIs) across diverse tumour types. A higher TMB level corresponds to an increased production of tumour neoantigens, thereby enhancing the likelihood of eliciting an immune response within the body [31].

**Figure 2:**
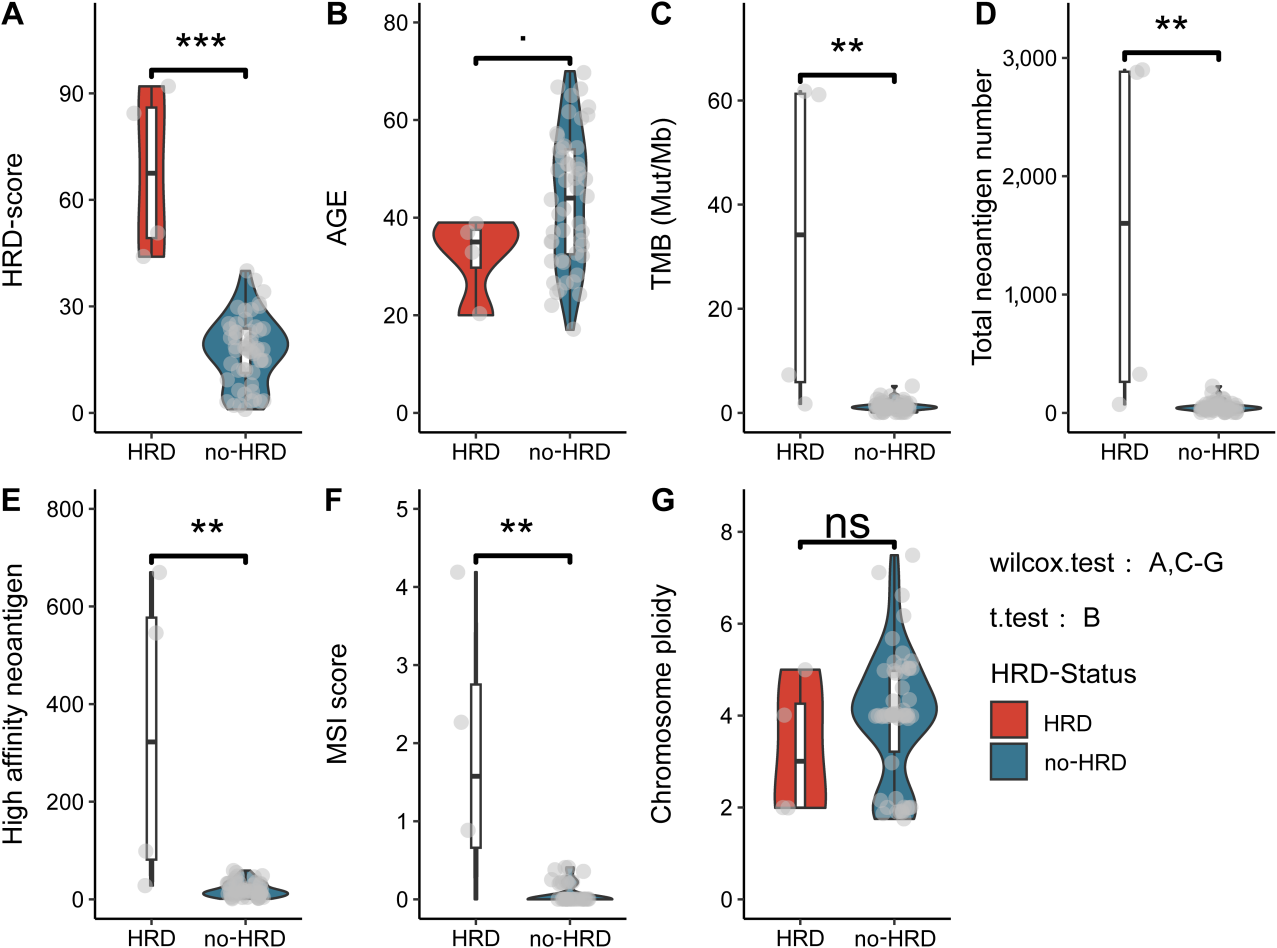
Differences in HRD score, TMB, TNB, and other indicators among different HRD statuses in the Zhujiang cohort. The boxplots illustrate the variations in each indicator across different HRD statuses in the Zhujiang cohort, specifically (A) HRD score, (B) age of patient (years), (C) tumour mutational burden (per MB), (D) TNB, (E) high affinity neoantigen, (F) MSI score, and (G) chromosome ploidy. The *t* test was employed if the data followed a normal distribution; otherwise, the Wilcoxon rank‐sum test was utilised. HRD score, homologous recombination deficiency score; HRD, homologous recombination deficiency; MSI score, microsatellite instability score; no‐HRD, without homologous recombination deficiency; t.test, Student's *t* test; total_per MB, total number of mutations per megabase (Mut/Mb) of DNA; wilcox.test, Wilcoxon rank‐sum test (Student's *t* test, Wilcoxon rank‐sum test, ***p < 0.001; **p < 0.01; * p < 0.05; *p* < 0.1).

To investigate the potential of ICIs as a treatment for HRD‐positive NPC, we calculated the differences in HRD score, age, TMB, TNB, high‐affinity neoantigen, MSI score, and chromosome ploidy in three cohorts according to HRD status grouping (HRD versus no‐HRD). The relevant statistical results of the Zhujiang cohort are shown in Figure 2. The statistical findings of the Singapore and Hong Kong cohorts can be found in supplementary material, Figures S3 and S4, respectively. Supplementary material, Tables S2–S4 present the clinical statistical data between different HRD statuses across the three cohorts, including gender, clinical stage, human leukocyte antigen (HLA)‐I molecular status, and other clinical characteristics. Our analysis revealed that individuals in the HRD group tended to be diagnosed at a younger age. Furthermore, the HRD group exhibited significantly higher levels of TMB, TNB, and high‐affinity neoantigens than the no‐HRD group.

Unfortunately, due to the limited sample size within a single cohort, the statistical analysis of MSI score and chromosome ploidy between different HRD statuses did not yield significant results. This outcome necessitates further exploration in a larger cohort to ascertain conclusive findings.

### The correlation between HRD status and prognosis in NPC

To determine whether HRD status has prognostic significance in NPC, we first applied univariate Cox proportional survival regression analysis in the Zhujiang cohort and found that there was no statistically significant correlation between HRD status and RFS [hazard ratio (HR), 2.48; 95% confidence interval (CI), 0.75–8.25; *p* = 0.14]. Given the potential for inconsistency in RFS between the primary and relapsed populations within the Zhujiang cohort, we conducted separate multivariate Cox proportional survival regression analyses for both the relapsed population and the total population.

**Figure 3:**
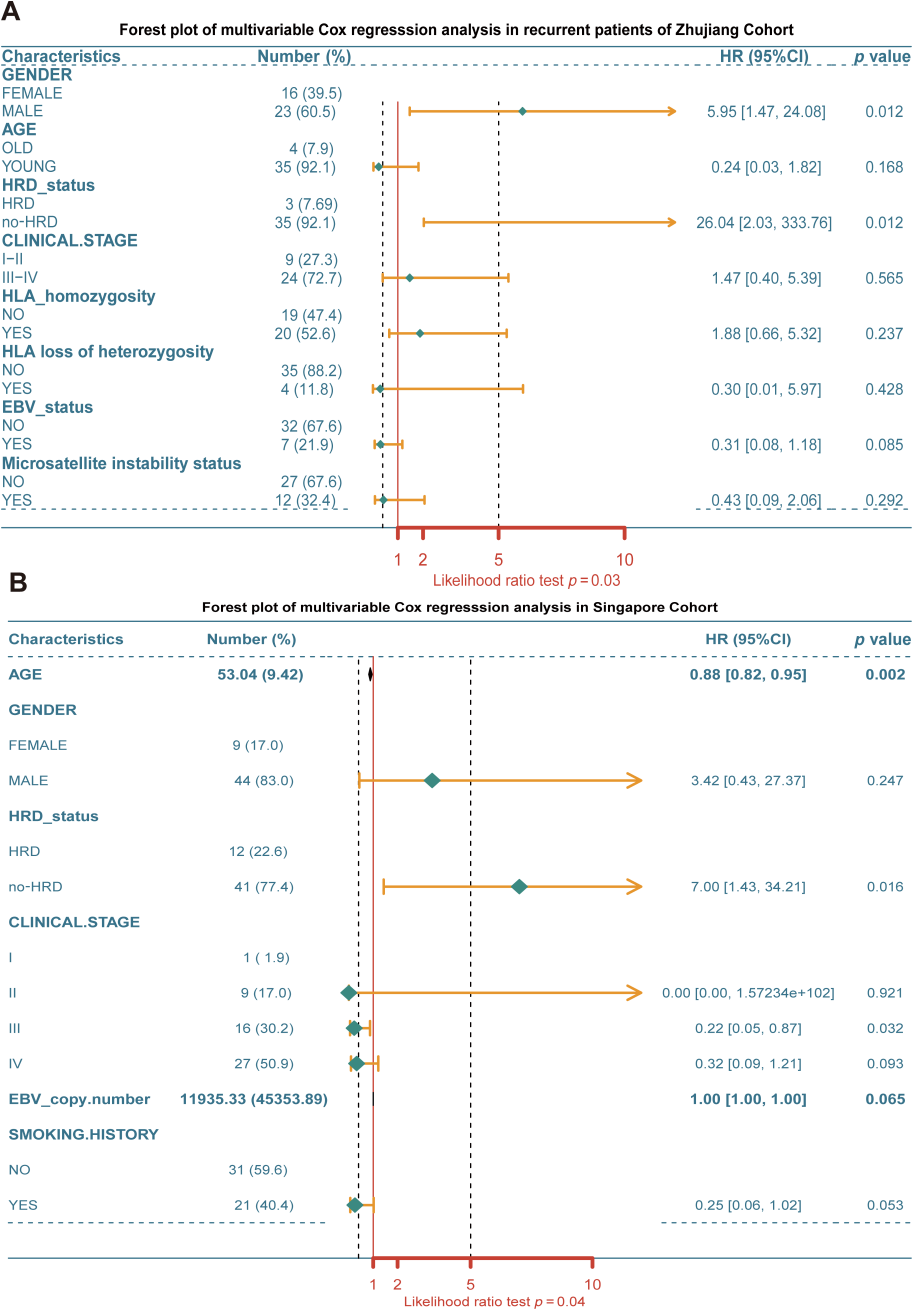
Results of multivariate Cox proportional survival regression analysis. Forest plots of multivariate cox proportional survival regression analysis in (A) the Zhujiang recurrent population and (B) the Singapore cohort. EBV, Epstein–Barr virus; HLA, human leukocyte antigen; HRD, homologous recombination deficiency; no‐HRD, without homologous recombination deficiency. Symbols: old = 60 or more for age; young = less than 60 for age.

First, we conducted multivariate Cox proportional survival regression analysis on 38 relapsed cases from the Zhujiang cohort. The analysis, after adjusting for age, sex, stage, and other factors, revealed that HRD status (HR, 26.03; 95% CI, 2.03–333.76; *p* = 0.012; Figure 3A) could serve as an independent prognostic factor in NPC and the no‐HRD group exhibited a higher risk of relapse. Subsequently, the result of multivariate Cox regression analysis that included all patients from the Zhujiang cohort was HR, 15.29; 95% CI, 1.56–150.07; *p* = 0.019; supplementary material, Figure S5. In the Singapore cohort, the application of multivariate Cox proportional survival regression analysis revealed a consistent result, indicating an increased risk of mortality in the no‐HRD group (HR, 7; 95% CI, 1.43–34.21; *p* = 0.016; Figure 3B).

Due to inconsistencies in the library preparation kits, sequencing platforms, and sequencing depths of the three cohorts, it was difficult to perform a combined analysis of the cohorts. Furthermore, the known clinical data (e.g. the Hong Kong cohort did not provide PFS) is not equivalent. After a comprehensive review of pertinent literature and in‐depth deliberation, we were uncertain whether the combined cohorts could be analysed collectively for survival statistics. Consequently, we did not perform a combined cohort analysis here.

Considering that the majority of recommended therapies for NPC involve the utilisation of platinum, radiotherapy, and other agents causing DNA damage, we hypothesised that individuals with HRD may exhibit heightened sensitivity to such treatment modalities, thereby resulting in a more favourable prognosis. Consequently, it is imperative to prioritise assessing HRD status during the management of NPC.

### HRD tumour tissue tends to be a non‐inflammatory immune microenvironment

**Figure 4:**
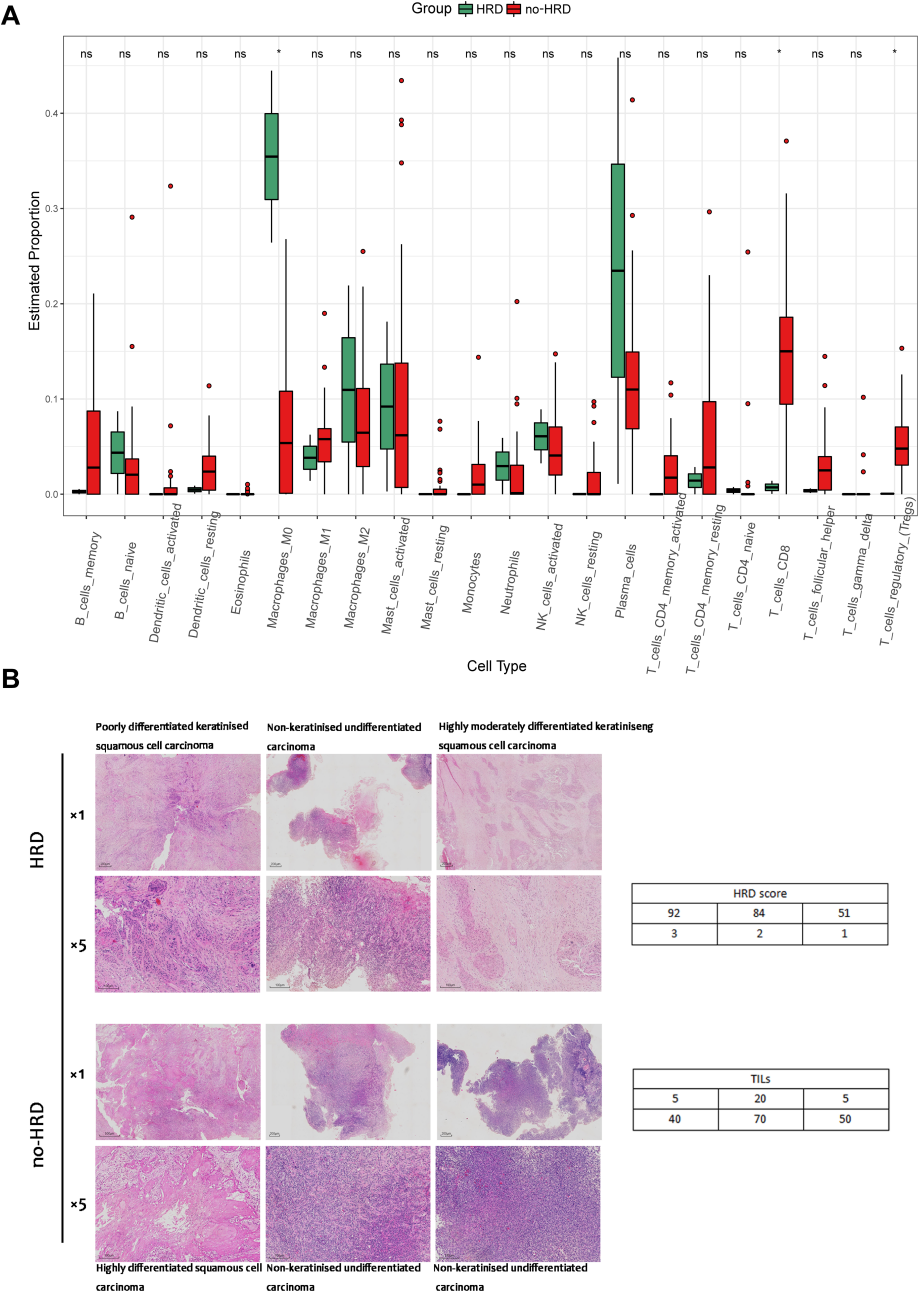
Profiling tumour‐infiltrating immune cells with CIBERSORT and representative HE staining images between the HRD and no‐HRD groups. (A) The results of CIBERSORT. M0 macrophages were significantly higher in HRD samples; CD8+ T cells, regulatory T cells (Tregs) were significantly higher in no‐HRD samples; and resting CD4+ memory T cells, activated CD4+ memory T cells also tended to be lower in HRD samples. (B) Representative HE staining images of the three patients with the highest (>42) and the three patients with the lowest HRD scores. The tables on the right show the specific HRD score and the percentage of HE‐stained TILs scored according to the recommendations of the International TILs Working Group. HE, haematoxylin eosin; HRD, homologous recombination deficiency; no‐HRD, without homologous recombination deficiency; TIICs, tumour infiltrating immune cells.

Of the 58 samples from the Zhujiang cohort, 41 were successfully subjected to RNA sequencing analysis. In contrast, the remaining samples were excluded from the analysis due to improper preservation or unsatisfactory RNA quality control. The ratio of HRD to no‐HRD among the 41 samples was 2:39. The immune infiltration of the samples was assessed using the CIBERSORT algorithm [19]. Our analysis revealed that the proportion of M0 macrophages was significantly higher in HRD samples, whereas CD8+ T cells and regulatory T cells (Tregs) were significantly higher in no‐HRD samples. Additionally, resting CD4+ memory T cells and activated CD4+ memory T cells tended to be lower in HRD samples (Figure 4A). For further characterisation of tumour infiltrating lymphocytes (TILs) in different HRD statuses, we chose the 10 highest HRD score HE‐stained slides we could find and the lowest HRD score HE‐stained slides as representative samples for re‐sweeping. We assessed the percentage of HE‐stained TILs as recommended by the International TILs Working Group (https://www.tilsinbreastcancer.org/) [20]. While correlating the TIL values with the HRD score values, we found that the HRD score and the infiltration ratio of TILs showed a significant negative correlation (*R* = −0.51, *p* = 0.021, Spearman); the specific correlation analysis values are shown in supplementary material, Table S5. We selected the HE staining images of the three patients with the highest (>42) and lowest HRD scores as the representative images to be shown in Figure 4B. These findings suggest that the HRD status may impact immune infiltration in NPC, with HRD‐positive tumour tissues tending to exhibit a more non‐inflammatory immune microenvironment.

Previous research has demonstrated the efficacy of high TMB and an inflammatory tumour immune microenvironment as biomarkers for ICIs [32]. Consequently, additional investigation and deliberation are required to determine the suitability of immunotherapy for HRD‐positive NPC patients.

### Differential analysis and pathway enrichment analysis

**Figure 5:**
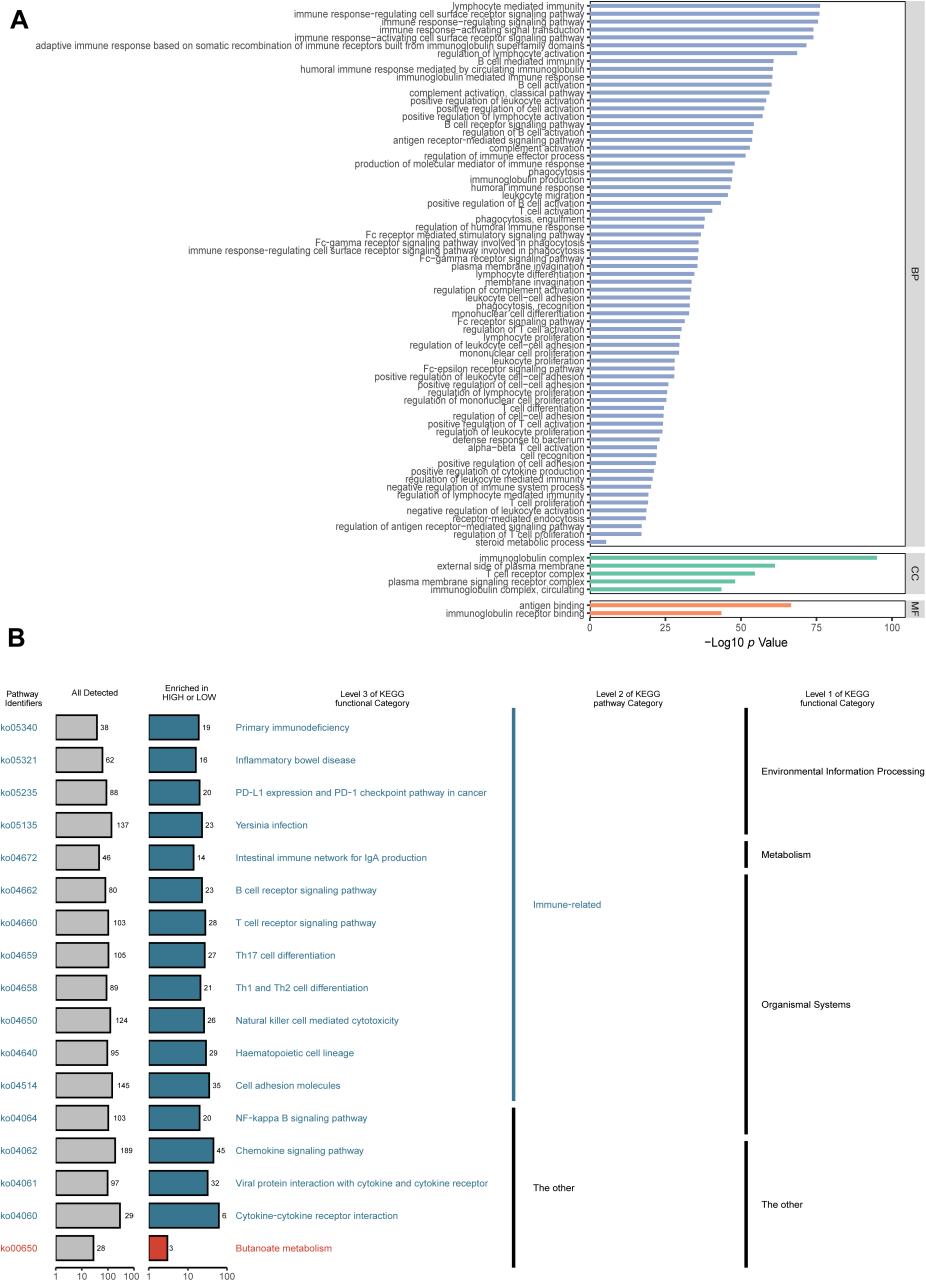
Gene ontology (GO)/Kyoto Encyclopedia of Genes and Genomes (KEGG) enrichment analysis between the HRD and no‐HRD groups carried out using R package clusterProfiler. (A) Enrichment analysis of GO terms for differential genes classified into three categories: biological process (GO‐BP), cell component (GO‐CC), molecular function (GO‐MF). (B) Enrichment analysis of KEGG terms for differential genes classified into two parts: immune‐related and other.

We conducted differential and pathway enrichment analyses to investigate further the distinctions between the HRD and the no‐HRD groups. The logFC (log2 fold change) cut‐off was set at 1.5, and a *p* value of 0.05 was utilised in the differential analysis using DEseq2 (supplementary material, Figure S6A). Subsequently, we conducted GO and KEGG enrichment analysis on the differentially expressed genes. The outcomes of the GO enrichment analysis indicated that the upregulated genes did not exhibit any discernible pathway enrichment. In contrast, the down‐regulated genes displayed a notable enrichment in immune‐related pathways, such as lymphocyte‐mediated immunity, T cell activation, and regulation of B cell activation (Figure 5A). The KEGG pathway enrichment analysis yielded comparable findings. These pathways included programmed death ligand 1 (PD‐L1) expression and the PD‐1 checkpoint pathway in cancer, the B or T cell receptor signalling pathway, Th1 and Th2 cell differentiation, etc. (Figure 5B).

We further carried out gene set enrichment analysis (GSEA) with HALLMARK and REACTOME‐related pathways on the data (supplementary material, Figure S6B,C). Similarly, immune‐related pathways such as IL6_JAK_STAT3_SIGNALLING and PD_1_SIGNALLING were significantly enriched in the no‐HRD group compared with the HRD group (see, in particular, supplementary material, Figure S6B,C).

The pathway enrichment analysis results indicated that HRD tumour tissue was in a microenvironment of immunosuppressive ‘cold tumour’, which is consistent with the results of CIBERSORT. However, previous studies have predominantly characterised NPC as a ‘hot tumour’ type with an inflammatory microenvironment [33]. Consequently, it is necessary to investigate and validate any potential direct or indirect association between HRD status and immune infiltration and elucidate the specific underlying mechanisms.

### The downregulation of BRCA1 expression enhances the sensitivity of NPC cells to radiotherapy and chemotherapy

**Figure 6:**
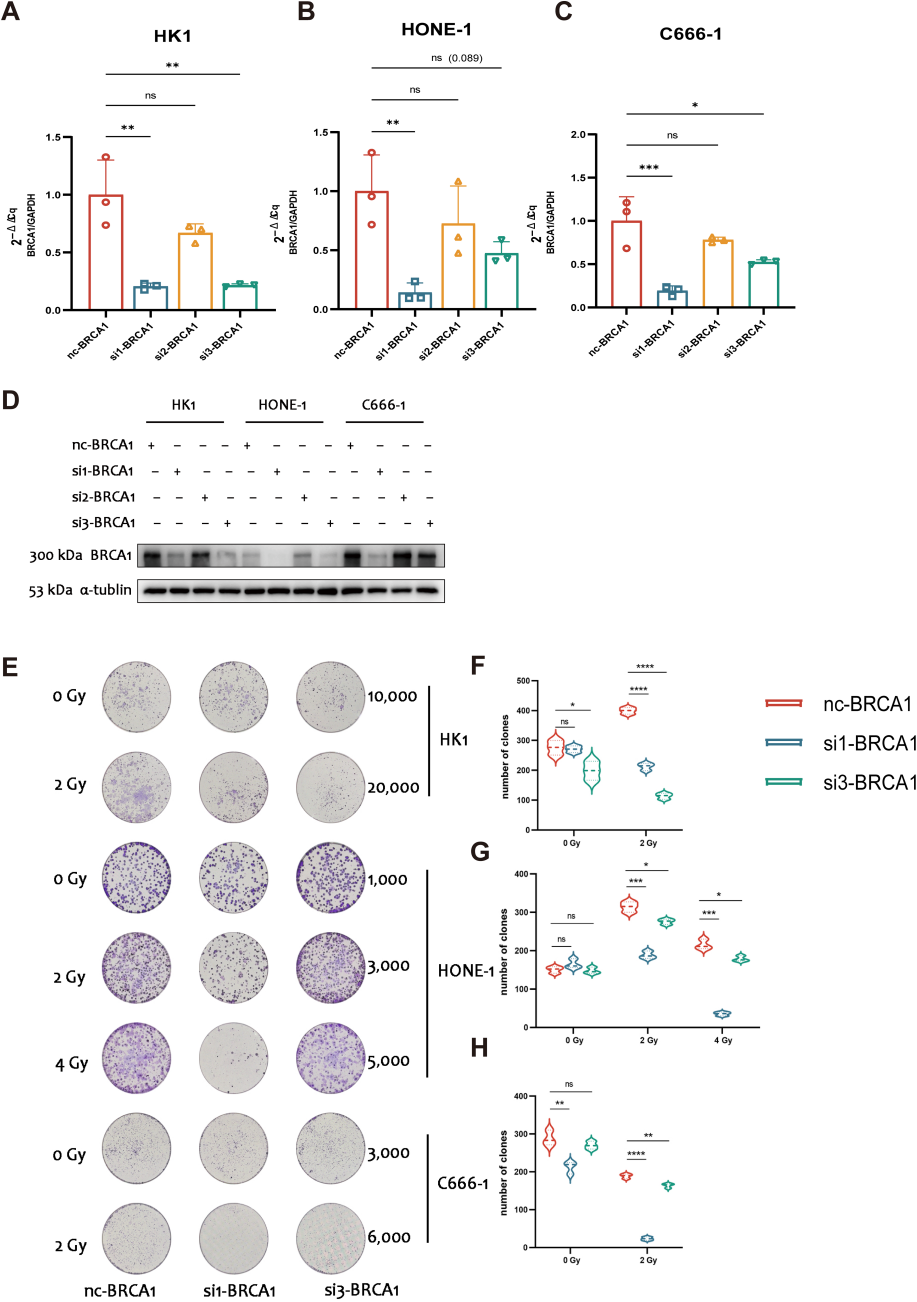
Validation of BRCA1 knockdown levels and demonstration that BRCA1 knockdown makes NPC cells more sensitive to radiotherapy. (A–D) Knockdown of BRCA1 at (A–C) mRNA and (D) protein levels using three small, interfering RNAs and validation of efficiency with RT‐qPCR and western blotting in HK1, HONE‐1 and C666‐1 cell lines. (E–H) Results of clone formation experiments (E) and statistics on the number of clones (F–H). The experiment was independently repeated three times. Student's *t* test, Wilcoxon rank‐sum test, ****p < 0.0001; ***p < 0.001; **p < 0.01; *p < 0.05; ns, not significant.

Based on our discovery that HRR status was linked to a relatively favourable prognosis in NPC, we hypothesised that individuals with HRD would exhibit heightened sensitivity to chemoradiotherapy. To substantiate this conjecture, we conducted *in vitro* validation using three NPC cell lines, namely HK1, HONE1, and C666‐1. Our focus was on BRCA1, a pivotal protein within the HRR pathway. By employing small, interfering RNA (si‐RNA) to knock down BRCA1 expression in the three NPC cell lines (Figure 6A–D), we observed alterations in cellular responsiveness to radiotherapy and cisplatin.

First, the clone formation assay was employed to assess the radiosensitivity of cells by comparing the number of clones formed after exposure to 0 or 2 Gy of radiation. The findings indicated a significant decrease in clones formed in the knockdown groups (si1‐BRCA1 and si3‐BRCA1) compared with the control group (nc‐BRCA1) following 2 Gy irradiation. Additionally, for the relatively radiation‐resistant HONE‐1 cells, the number of clones formed after 4 Gy irradiation was also examined, and the results were consistent with those observed under 2 Gy irradiation (Figure 6E–H).

Subsequently, we investigated the variations in cellular responsiveness to cisplatin after the knockdown of BRCA1 expression through si‐RNA. Our findings revealed a significant increase in cellular sensitivity to cisplatin following BRCA1 knockdown. The IC_50_ values for nc‐BRCA1, si1‐BRCA1, and si3‐BRCA1 were determined to be 1.320 (95% CI, 1.182–1,472), 0.9252 (95% CI, 0.8506–1.005), and 0.7339 (95% CI, 0.6723–0.8001), respectively, in HK1 (supplementary material, Figure S7A,B). In HONE‐1, the IC50 of nc‐BRCA1, si1‐BRCA1 and si3‐BRCA1 were 2.411 (95% CI, 2.163–2.689), 1.465 (95% CI, 1.320–1.625) and 1.841 (95% CI, 1.653–2.051), respectively (supplementary material, Figure S7C,D). In C666‐1, the IC50 of nc‐BRCA1, si1‐BRCA1 and si3‐BRCA1 were 4.886 (95% CI, 4.451–5.368), 1.485 (95% CI, 1.250–1.757) and 2.389 (95% CI, 2.218–2.573), respectively (supplementary material, Figure S7E,F).

In conclusion, it can be inferred that NPC cells exhibit heightened sensitivity to chemoradiotherapy *in vitro* when the expression of BRCA1 is comparatively low or dysfunctional or when the cellular HRR function is relatively impaired. This observation partially elucidates why HRD patients displayed a relatively favourable prognosis.

## Discussion

There are few studies on homologous recombination‐related genomic instability in NPC. HRD is common in ovarian, breast, prostate, and pancreatic cancers and has been extensively studied [34]. A study on patients with locally advanced NPC in Southeastern Europe (SEE) who underwent radiotherapy and chemotherapy revealed a notable occurrence of somatic *BRCA1* mutations in SEE NPC. Furthermore, it was observed that *BRCA1* mutations appeared to be an unfavourable prognosticator only in tumours of intermediate stability [35]. These results suggest that the association of genomic stability or HRD with prognosis in NPC remains unclear. Therefore, we evaluated genomic instability or HRD in a different and more common manner to analyse the impact of genomic instability or HRD on the prognosis of NPC patients comprehensively.

In an HNSCC study, tumours with high HRD scores were associated with relatively poor survival and tended to have a non‐inflamed tumour microenvironment [36]. However, our analysis revealed that HRD patients in NPC had a reduced risk of recurrence and mortality, indicating a protective effect. It is important to note that head and neck cancer encompasses diverse tumour types, exhibiting commonalities and specificities among individual cancer subtypes. So, a separate analysis of NPC is warranted.

Currently, the predominant approach for managing NPC patients involves the integration of radiotherapy and chemotherapy as a comprehensive treatment. Platinum‐based drugs and other agents that specifically target DNA damage are extensively employed in treating NPC [1]. Pertinent investigations have demonstrated that individuals with HRD in ovarian, breast, prostate, and pancreatic carcinomas exhibit heightened sensitivity to platinum compounds and PARPi; furthermore, certain studies have indicated that HRD‐positive patients experience more favourable prognoses than HRD‐negative patients [3, 4, 5, 6, 7, 8]. So, identifying patients with HRD holds significant value in informing the administration of platinum and PARPi therapies in NPC, facilitating the selection of optimal treatment strategies, and predicting treatment response and prognosis. Consequently, employing HRD status as a biomarker for patient stratification and subsequent personalised treatment selection would yield additional advantages for cancer patients.

PARPi exhibit selective toxicity towards cells with HRD, primarily through the synthetic lethal effect [10]. Although PARPi are not currently employed as a routine treatment for NPC, ongoing clinical trials such as NCT04978012, NCT05162872, and NCT04825990 investigating their application in NPC indicate a growing interest in the therapeutic potential of PARPi. The findings of this study further support the rationale for utilising PARPi in the treatment of NPC.

It has been postulated that varying immune microenvironments may result in divergent reactions to PARPi and Immune checkpoint blockades [37]. Formerly, it was believed that NPC possessed a distinctive immune landscape and was a highly immune inflammatory tumour, rendering it well‐suited for immunotherapy [33]. Our findings indicated that the immune microenvironment of the HRD group tended to exhibit characteristics of a ‘cold tumour’, with a notable decrease in T cell infiltration. This result was derived from a relatively limited sample size, so it is somewhat controversial, and further analysis of the relationship between HRD status and TMB, pathological type, and immune infiltration in NPC will be needed in the future when more sequencing samples are collected.

Empirical evidence indicates that HRD‐positive tumours exhibit elevated levels of TILs, TMB, and the expression of PD‐L1 [37, 38, 39]. Conversely, several studies have demonstrated a correlation between HRD deficiency and a relatively non‐inflamed tumour microenvironment [36, 40]. A comprehensive examination of HRD scores in relation to 32 solid tumours within the TCGA project revealed that only a few cancer types exhibited a correlation between high HRD scores and an immune‐activated tumour microenvironment [41]. This finding suggests that the association between HRD and the tumour immune microenvironment varies across different cancer types, necessitating further extensive investigation. Additionally, given the limited sample size employed in this study, additional samples and more comprehensive research are imperative for validation purposes.

Furthermore, we performed Fisher's exact test (*p* = 0.05) for HRD status and pathology type (mainly keratinisation status) in the Zhujiang cohort. We found using this analysis that, with a limited sample size, keratinisation predominated among HRD‐positive patients, with a percentage of 50. This needs to be further verified with more NPC samples in the future.

We also analysed the status of HLA‐I molecules in three cohorts, specifically focusing on HLA‐I homogeneity and LOH, the presence of the APOBEC mutation signature (a widely observed characteristic in various cancer types with certain clinical prognostic implications), microsatellite instability (MSI, which serves as a molecular indicator of defects in mismatch repair systems), and chromosomal ploidy (abnormal ploidy status being prevalent in malignant tumours). These features above belong to a series of genome‐level alterations that possess prognostic or predictive value in some or individual tumours.

In summary, this study investigated the clinical implications, molecular features, and immune profiles associated with HRD status in NPC using a local cohort and two publicly available datasets. First, our results provide additional evidence supporting the notion that HRD status significantly impacts the prognosis of NPC patients. Second, we observed that HRD‐positive tumours exhibit elevated levels of TMB and TNB and tend to a non‐inflammatory tumour immune microenvironment, representing the first report of such findings in NPC patients from Southeast Asia. Third, our research highlighted the presence of a specific subset of HRD in NPC, suggesting the potential efficacy of therapeutic agents targeting HRR mechanisms, such as platinum and PARPi. Moreover, our study provides evidence that silencing BRCA1 notably augments the susceptibility of NPC cells to chemoradiotherapy *in vitro*.

This study is subject to certain limitations, primarily due to the low sample size of a single cohort, necessitating a larger sample size to confirm further the prognostic significance of HRD in NPC. We did not calculate NPC‐specific HRD score cut‐off for some objective reasons, which we will do in the future when we have larger NPC sequencing cohorts and more sufficient clinical information. Additionally, more mRNA sequencing data from tumour samples are required to delve deeper into the immune infiltration of HRD tumour tissues. While the promotion and application of HRD detection in NPC shows promise, there remains a long way to go.

In conclusion, patients diagnosed with HRD in NPC have a comparatively favourable prognosis, and they may exhibit heightened sensitivity to drugs targeting HRR systems (platinum, PARPi, etc.). Whether these patients are suitable for ICIs needs further in‐depth study.

## Author contributions statement

JZ and XZ conceived and supervised the study. XZ, HY and SZ wrote the manuscript. XZ, YS, WZ and PL conducted statistical analysis and visual representations. The manuscript was reviewed by all authors.

## Supporting information


Supplementary materials and methods

**Supplementary links.** Specific analysis tools and accessible source links

**Figure S1.** Plot of all germline mutations in 17 major genes of the HRR pathway across the three cohorts
**Figure S2.** Plot of all somatic mutations in 17 major genes of the HRR pathway across the three cohorts
**Figure S3.** Differences in HRD score, TMB, TNB, and other indicators among different HRD statuses in the Singapore cohort
**Figure S4.** Differences in HRD score, TMB, TNB, and other indicators among different HRD statuses in the Hong Kong cohort
**Figure S5.** Results of multivariate Cox proportional survival regression analysis in the total population of the Zhujiang cohort
**Figure S6.** Plots of differential analysis and results of GSEA
**Figure S7.** Knockdown of BRCA1 makes NPC cells more sensitive to chemotherapy (cisplatin)

**Table S1.** Tumour purity/cellularity and ploidy data for all three cohorts

**Table S2.** Clinical characteristics of the Zhujiang cohort according to HRD status
**Table S3.** Clinical characteristics of the Singapore cohort according to HRD status
**Table S4.** Clinical characteristics of the Hong Kong cohort according to HRD status

**Table S5.** Detailed Zhujiang cohort clinical information

## Data Availability

The WES data for the Singapore and Hong Kong cohorts were obtained from the SRA database, specifically accession numbers SRP035573 (Singapore) and SRA288429 (Hong Kong), respectively. The clinical data for these cohorts were obtained from the respective original articles [11, 12]. The corresponding author can provide all data from the Zhujiang cohort upon reasonable request.
