# Supplementary material for: Homologous recombination deficiency (HRD) is associated with better prognosis and possibly causes a non‐inflamed tumour microenvironment in nasopharyngeal carcinoma
Source: J Pathol Clin Res. 2024 Aug 5;10(5):e12391. doi: 10.1002/2056-4538.12391 (PMC11300531; doi:10.1002/2056-4538.12391)
Supplement: Supplementary file 1 — Supplementary materials and methods [file CJP2-10-e12391-s005.pdf]

Homologous recombination deficiency (HRD) is associated with better prognosis and possibly causes a non-inflamed tumour microenvironment in nasopharyngeal carcinoma

X Zhou *et al., J Pathol Clin Res*, https://doi.org/10.1002/2056-4538.12391

Supplementary materials and methods

Reference numbers refer to the list in the main paper

Bulk RNA-seq

Total RNA was extracted from clinical specimens that were freshly quick-frozen using trizol. The extracted Total RNA was then subjected to analysis for purity using NanoDrop™ One (Thermo, ND-ONE-W), concentration using Qubit™ 3 Fluorometer (Thermo, Qubit 3.0), and integrity using Agilent 4200 TapeStation System (Agilent, Agilent 4200). Subsequently, the samples that met the quality control criteria underwent library construction and sequencing. A total of 1 microgram (1ug) of RNA was extracted and subjected to mRNA enrichment using the VAHTS® mRNA Capture Beads (Vazyme, N401-02) kit. Subsequently, the enriched mRNA was fragmented using the VAHTS® Universal V8 RNA-seq Library Prep Kit for Illumina (Vazyme, NR605-02) kit. Following reverse transcription and PCR amplification of the resulting cDNA products, the library was sequenced using the NovaSeq 6000 instrument and the corresponding NovaSeq S4 reagent kit.

RNA-seq analysis

The raw data were processed using Fastp version 0.12.4 [21] for filtering and quality control. Subsequently, the aligned raw data were mapped to the human reference

genome (GRCh38) using HISAT2 version 2.2.1 [22]. The quantification and synthesis of the counts expression matrix were performed using featureCounts in Rsubread_2.0.0 [23]. Differential expression analysis was conducted using DESeq2 version 1.28.1 [24]. The subsequent analysis utilised TPM normalised data.

Estimation of the immune cell population

First, 22 immune cell infiltration differentials were assessed using the CIBERSORT R package in Rstudio employing the default parameters [19], and we evaluated the TILs from HE-stained sections according to the recommendations of the International TILs Working Group [20].

Gene Ontology (GO) and Kyoto Encyclopedia of genes and genomes pathway enrichment analysis (KEGG)

GO/KEGG functional enrichment analysis was completed using R Package clusterProfiler version 3.16.1 [25], and the cut-off value for P-value was taken as 0.05. Significantly enriched GO pathways were categorised into three groups in order of biological process (BP), cellular component (CC), and molecular function (MF). KEGG-enriched pathways were categorised into two groups according to whether they were immune-related or not, in order of Immune-related and the other.

Gene set enrichment analysis (GSEA)

The Gene Set Enrichment Analysis (GSEA gsea-msigdb.org) assesses the distribution

pattern of all genes within a predetermined gene set (such as a pathway) in a gene list sorted based on phenotypic relevance. This analysis aims to ascertain how much this gene set contributes to the observed phenotype. The differential analysis results were sorted based on the logFC, and the GSEA pathway enrichment analysis was conducted using the R packages "GSEAbase" and "clusterProfile" to calculate the enrichment score (ES) and p-value. This study primarily focused on performing enrichment analysis on the HALLMARKS and REACTOME gene sets. Pathways that exhibited significant enrichment in either the HRD or the no-HRD group will be presented.

Determination of HLA-I status

We primarily examined two facets of human leukocyte antigen (HLA)-I molecules: HLA-I germline homogeneity and loss of heterozygosity (LOH). To evaluate HLA-I germline homogeneity, we utilised the bam files derived from the alignment of whole exome sequencing (WES) data from peripheral blood as input files for Polysolver. We employed the parameters (arguments: Asian 1 hg19 STDFQ 0) to obtain the 4-digit HLA-I genotypes for each individual. Patients who exhibited the same genotypes for both alleles at any of the HLA-A, HLA-B, and HLA-C loci were classified as having HLA-I germline homogeneity. In contrast, those with differing genotypes were classified as having HLA-I germline heterogeneity. To assess the loss of heterozygosity (LOH) in human leukocyte antigen class I (HLA-I), we employed the LOHHLA tool [26], utilising the bam files derived from aligning tumour tissues and paired peripheral blood.

Additionally, the tumour purity and ploidy, determined by sequenza, and the specific HLA-I genotype from hla-polysolver were utilised as input files. If the P-Val_unique value was less than 0.05, it indicated LOH in the respective allele, thereby classifying the sample as having HLA-I LOH. Conversely, if the P-Val_unique value was greater than or equal to 0.05, the sample was classified as HLA-I non-LOH.

The prediction and calculation of neoantigens by Pvacseq

The Pvacseq pipeline (Version: 2.0.4) was employed to forecast neoantigens or tumour-specific mutant peptides [27]. The NetMHC PickPocket algorithm was utilised to anticipate HLA-I associated neoantigens with predicted epitope length of 8, 9, 10, or 11. the lower of the two algorithms in terms of wild type (WT) IC50 score was used as a criterion of statistical analysis. Epitopes where the mutant allele has ic50 binding scores below 500, were considered high affinity neoantigens.

The judgement of APOBEC mutation characteristics

The APOBEC family, which consists of Apolipoprotein B mRNA editing enzyme, catalytic polypeptide-like, is a group of cytidine deaminases that has been conserved throughout evolution. Human cancer genomes frequently exhibit APOBEC mutation patterns [28]. The Maftools trinucleotideMatrix function is employed to assess the Enrichment score of samples for APOBEC mutations and determine if such mutations are enriched.

Microsatellite Instability(MSI) score or status

The MSIsensor-pro software [29] was employed to assess the Microsatellite Instability (MSI) in cancer patients by utilising next-generation sequencing data, thereby obtaining the MSI status score. The microsatellite sites files were obtained using GRCh38 as the reference genome. In our research, the MSI score was directly employed for statistical analysis.

Cell lines and culture conditions

The NPC cell lines HK-1, C666-1, and HONE-1 were obtained from Guangzhou Qunxian Biotechnology Co., Ltd. These cell lines were cultured in Roswell Park Memorial Institute (RPMI) 1640 medium (KeyGEN BioTECH, KGM31800-500, Nanjing, China) supplemented with 10% fetal bovine serum (FBS) (Procell, 164210-50, Wuhan, China) and 1% penicillin/streptomycin at a temperature of 37 °C in a humidified atmosphere containing 5% CO2. A Short tandem repeat (STR) authentication report can be provided to verify these cell lines. Experiments were conducted using exponentially growing cells, and regular mycoplasma testing was performed on all cells.

Calculation of IC50 by Cell Counting Kit-8 (CCK-8) In vitro

For the CCK-8 assay, NPC cells that had been transfected were plated in a 96-well plate at a density ranging from 4,000 to 10,000 cells per well. Following a 24-hour incubation period, the cells were exposed to a gradient of cisplatin concentrations (ranging from 0

to 160 μg/ml; Sigma-Aldrich, St. Louis, MO, USA) for 24 hours. Subsequently, the cells were incubated with 100 μL of fresh medium containing 10% CCK-8 reagent (APExBIO, USA) for 2 hours at 37 °C. The absorbance at a wavelength of 450 nm was measured using a microplate reader (BioTek, USA). The IC50 value was determined using GraphPad Prism 9 software (GraphPad Software, La Jolla, CA, USA).

Clonogenic formation assay

Different numbers of cells (1,000–20,000) were plated In 6-well plates. The cells were irradiated with a 6-MV Photon beam generated by a linear accelerator (Elekta, SYNERGY PL, Sweden) at a dose rate of about 614cGy/min (source-to-skin distance (SSD): 100 cm;). The colonies were fixed with 4% Paraformaldehyde for 15 minutes and then stained with 0.1% crystal violet for 30 minutes each.

Subsequently, the plates were washed with tap water and dried at room temperature. The number of clones was counted using ImageJ software, and we compared the number of clones in the nc-BRCA1 group and the si-BRCA1 groups at a dose of 2Gy to reflect alterations in cellular radiosensitivity. Additionally, for the HONE-1 cell line, which exhibits relatively higher resistance to radiation, the number of clones was also compared at a dose of 4 Gy.

Transfection of siRNA

The siRNA used in this study was obtained from Guangzhou HanYi Biosciences Inc. The specific target sequences for *BRCA1* are presented below:

5’-AAG GAA CCU GUC UCC ACA AAG UU-3’

5’-CAGCAGTTTATTACTCACTAA-3’

5’-CAGGAAATGGCTGAACTAGAA-3’

For nc-*BRCA1*

5’-UUCUCCGAACGUGUCACGUTT-3’

Transfection was conducted in six-well plates employing PepMute (SignaGen, SignaGen Laboratories, USA) small interfering RNA transfection reagent, following the guidelines provided by the manufacturer. The si-RNA transfection was carried out at a final concentration of 50nM.

Cell irradiation

Irradiation was delivered at room temperature with a 6-MV Photon beam generated by the linear accelerator (Elekta, SYNERGY PL, Sweden) at a dose rate of about 614 cGy/min. The bottom of the cell culture containers was coated with a 1cm thickness compensation glue. The source-to-skin distance was 100 cm.

Real-time quantitative PCR (RT-qPCR)

Total RNA was extracted from transfected nc-*BRCA1* cells and corresponding transfected si-*BRCA1* cells using the EzB rapid RNA extraction kit (EZBioscience, B0004DP, USA) following the manufacturer's instructions. The synthesis of cDNA from the RNA samples through reverse transcription was conducted using the EzB Color All-in-one Reverse Transcription Kit (with DNase) (EZBioscience, A0010CGQ,

USA). Subsequently, Real-time quantitative PCR (RT-qPCR) was conducted using 2× EZ Color SYBR Green qPCR Master Mix (ROX2 plus) (EZBioscience, RT3C, USA) on a BioRad instrument. *GAPDH* was utilised as a reference gene to standardise the relative mRNA expression, which was determined using the $2^{-\Delta\Delta Ct}$ method. The primers employed in this investigation were as follows:

BRCA1-F 5'-ACCTTGGAACTGTGAGAACTCT-3'

BRCA1-R 5'-TCTTGATCTCCCACACTGCAATA-3'

GAPDH-F 5'-GGAGCGAGATCCCTCCAAAAT-3'

GAPDH-R 5'-GGCTGTTGTCATACTTCTCATGG-3'

Protein extraction and western blot

Total protein was extracted using RIPA buffer (FUDE BIOLOGICAL TECHNOLOGY CO., LTD., Hangzhou, China) and quantified using the BCA method (KeyGEN BioTECH, KGM31800-500, Nanjing, China). The proteins were subsequently separated on 10% SDS–PAGE gels and transferred onto PVDF membranes. Following blocking with 5% nonfat milk, the membranes were incubated overnight at 4℃ with BRCA1 primary antibodies (Proteintech, Cat No. 22362-1-AP, Wuhan, China). The following day, the membranes underwent incubation with secondary antibodies labeled with HRP (CWBio, CW0103S, China) for 1 hour at room temperature. α-tubulin (Proteintech, Wuhan, Cat No.66031-1-Ig, China) was employed as an internal control.

The corresponding secondary antibody was Goat Anti-Mouse IgG, HRP Conjugated (CWBio, CW0102, China). The protein bands on the membranes were detected using a UVITEC authentic imaging system (Cambridge, UK).
