# Supplementary material for: Homologous recombination deficiency (HRD) is associated with better prognosis and possibly causes a non‐inflamed tumour microenvironment in nasopharyngeal carcinoma
Source: J Pathol Clin Res. 2024 Aug 5;10(5):e12391. doi: 10.1002/2056-4538.12391 (PMC11300531; doi:10.1002/2056-4538.12391)
Supplement: Supplementary file 2 — Supplementary links. Specific analysis tools and accessible source links [file CJP2-10-e12391-s004.pdf]

Homologous recombination deficiency (HRD) is associated with better prognosis and possibly causes a non-inflamed tumour microenvironment in nasopharyngeal carcinoma

X Zhou *et al.* *J Pathol Clin Res* https://doi.org/10.1002/2056-4538.12391

Supplementary Links

1. GATK

GATK (broadinstitute.org)

https://gatk.broadinstitute.org/hc/en-us

2. Fastp version 0.12.4

GitHub - OpenGene/fastp: An ultra-fast all-in-one FASTQ preprocessor (QC/adapters/trimming/filtering/splitting/merging...)

https://github.com/OpenGene/fastp

3. Burrows-Wheeler-Alignment Tool (BWA0.7.17-r1188)

Burrows-Wheeler Aligner - Browse Files at SourceForge.net

https://sourceforge.net/projects/bio-bwa/files/

4. ensembl-vep 104.3

Ensembl genome browser 112

http://www.ensembl.org/index.html

5. Maftools

maftools : Summarize, Analyze and Visualize MAF Files (bioconductor.org)

https://bioconductor.org/packages/release/bioc/vignettes/maftools/inst/doc/maftools.html

6. Google Cloud bucket

https://console.cloud.google.com/storage/browser/genomics-public-data/resources/broad/hg38/v0/

7. Sequenza 3.0.0

https://www.rdocumentation.org/packages/sequenza/versions/3.0.0

sequenza package - RDocumentation

8. HISAT2 version 2.2.1

https://daehwankimlab.github.io/hisat2/download/

Download | HISAT2 (daehwankimlab.github.io)

9. CIBERSORT R package

https://github.com/Moonerss/CIBERSORT

GitHub - Moonerss/CIBERSORT

10. GSEA

https://www.gsea-msigdb.org/gsea/index.jsp

GSEA (gsea-msigdb.org)

11. Polysolver

GitHub - sahilseth/polysolver

https://github.com/sahilseth/polysolver

12. LOHHLA

https://github.com/mskcc/lohhla

GitHub - mskcc/lohhla: Fork from https://bitbucket.org/mcgranahanlab/lohhla/src, modified for MSKCC needs

13. Pvacseq pipeline (Version: 2.0.4)

pVACseq — pVACtools 4.2.0 documentation

https://pvactools.readthedocs.io/en/latest/pvacseq.html

14. MSIsensor-pro

https://github.com/xjtu-omics/msisensor-pro

GitHub - xjtu-omics/msisensor-pro: Microsatellite Instability (MSI) detection using high-throughput sequencing data.

15. Singapore Cohort

SRP035573

https://www.ncbi.nlm.nih.gov/sra/?term=SRP035573

16. Hong Kong Cohort

SRA288429

https://www.ncbi.nlm.nih.gov/sra/?term=SRA288429
