# Supplementary material for: Homologous recombination deficiency (HRD) is associated with better prognosis and possibly causes a non‐inflamed tumour microenvironment in nasopharyngeal carcinoma
Source: J Pathol Clin Res. 2024 Aug 5;10(5):e12391. doi: 10.1002/2056-4538.12391 (PMC11300531; doi:10.1002/2056-4538.12391)
Supplement: Supplementary file 3 — Figure S1. Plot of all germline mutations in 17 major genes of the HRR pathway across the three cohorts Figure S2. Plot of all somatic mutations in 17 major genes of the HRR pathway across the three cohorts Figure S3. Differences in HRD score, TMB, TNB, and other indicators among different HRD statuses in the Singapore cohort Figure S4. Differences in HRD score, TMB, TNB, and other indicators among different HRD statuses in the Hong Kong cohort Figure S5. Results of multivariate Cox proportional survival regression analysis in the total population of the Zhujiang cohort Figure S6. Plots of differential analysis and results of GSEA Figure S7. Knockdown of BRCA1 makes NPC cells more sensitive to chemotherapy (cisplatin) [file CJP2-10-e12391-s001.pdf]

Homologous recombination deficiency (HRD) is associated with better prognosis and possibly causes a non-inflamed tumour microenvironment in nasopharyngeal carcinoma

X Zhou *et al. J Pathol Clin Res* https://doi.org/10.1002/2056-4538.12391

Supplementary Figures S1–S7

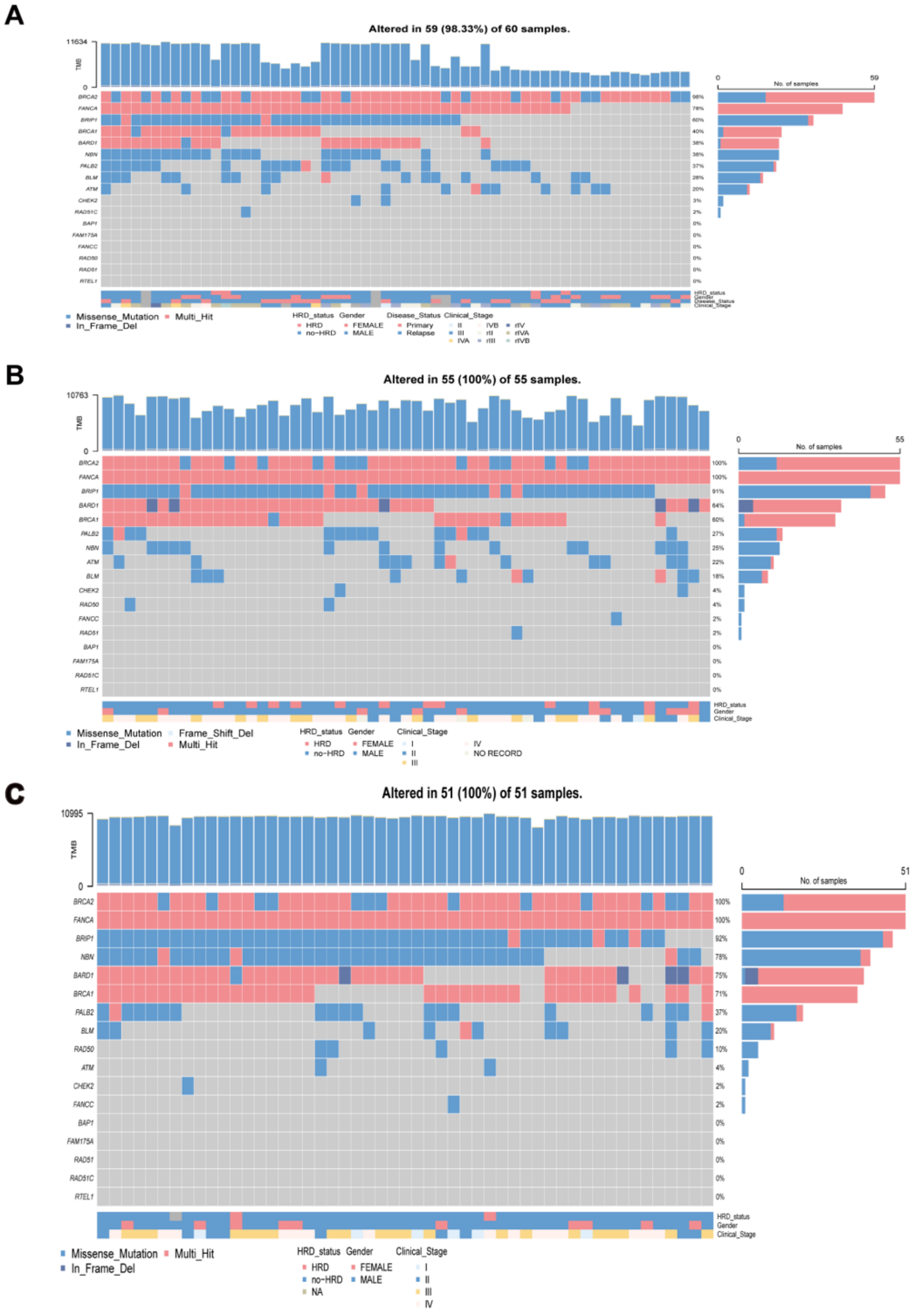

Figure S1. Plot of all germline mutations in 17 major genes of the HRR pathway across the three cohorts.

The 17 genes were as follows, *BRCA1, BRCA2, PALB2, ATM, BAP1, BARD1, BLM, BRIP1, CHEK2, FAM175A, FANCA, FANCC, NBN, RAD50, RAD51, RAD51C, RTEL1*; all mutations are shown without distinguishing clinical significance. (A) In the Zhujiang cohort, the top five mutated genes were ranked as follows: *BRCA2, FANCA, BRIP1, BRCA1,* and *BARD1*. (B) Similarly, in the Singapore cohort, the top five mutated genes were *BRCA2, FANCA, BRIP1, BARD1,* and *BRCA1*. (C) In the Hong Kong cohort, the top five mutated genes identified were *BRCA2, FANCA, BRIP1, NBN,* and *BARD1*.

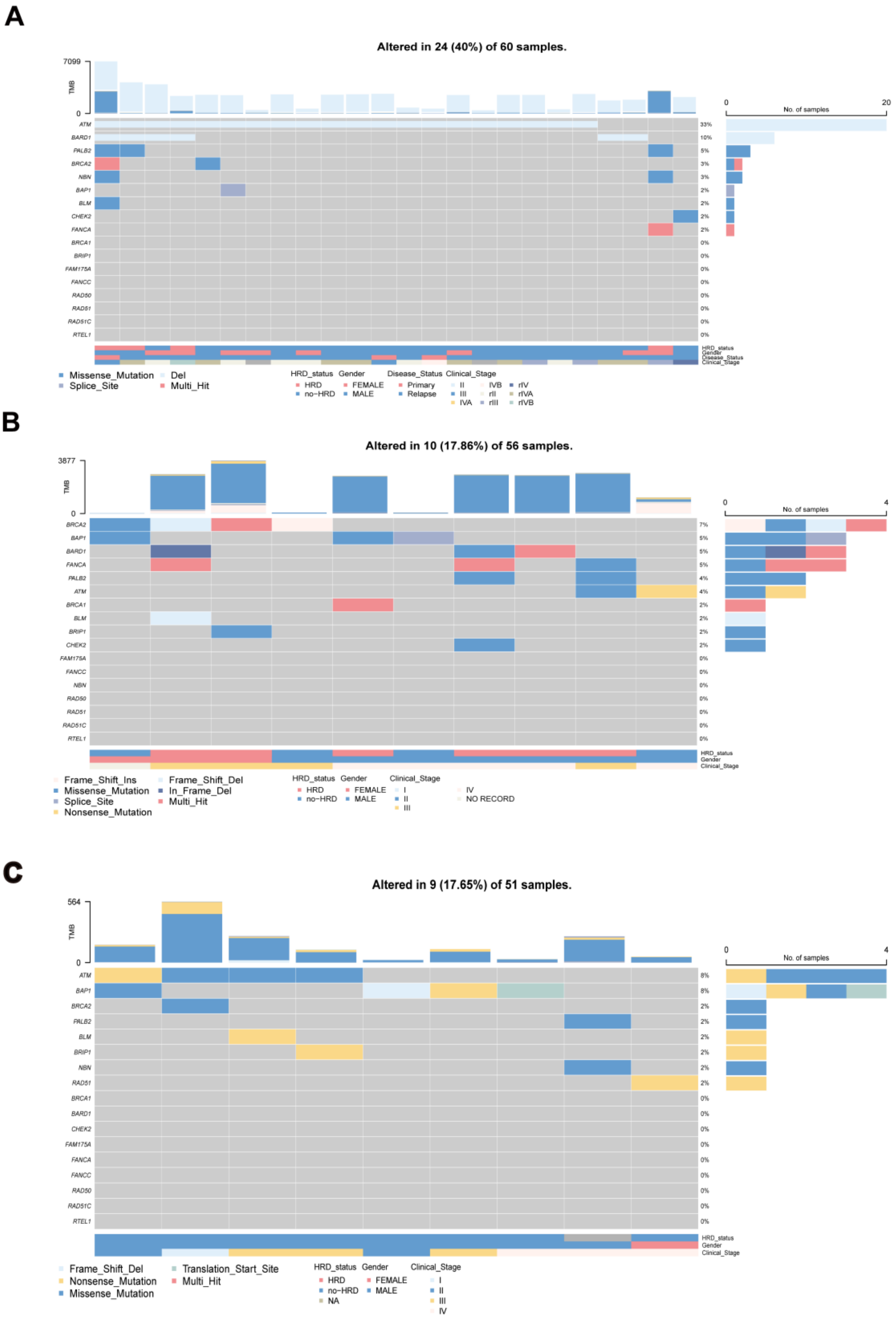

Figure S2. Plot of all somatic mutations in 17 major genes of the HRR pathway across the three cohorts.

(A) In the Zhujiang cohort, the top three mutated genes were ranked as follows: *ATM, BARD1, PALB2*. (B) Similarly, in the Singapore cohort, the top three mutated genes were *BRCA2, BAP1, BARD1*. (C) In the Hong Kong cohort, the top three mutated genes identified were *ATM, BAP1, BRCA2*.

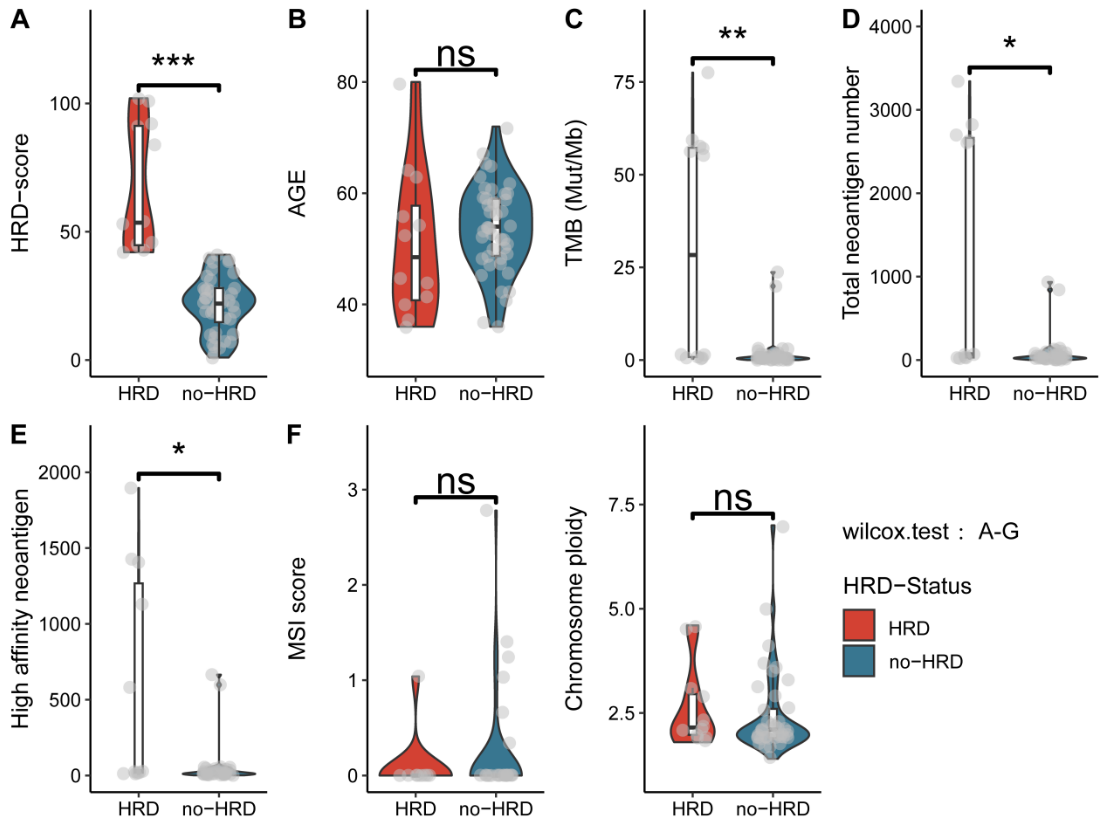

Figure S3. Differences in HRD score, TMB, TNB, and other indicators among different HRD statuses in the Singapore cohort.

The plots illustrate the variations in each indicator across different HRD statuses in the Singapore cohort, specifically (A) HRD score, (B) patient age, (C) tumour mutational burden (Mut/Mb), (D) total neoantigen number, (E) high affinity neoantigen, (F) MSI score, and (G) chromosome ploidy.

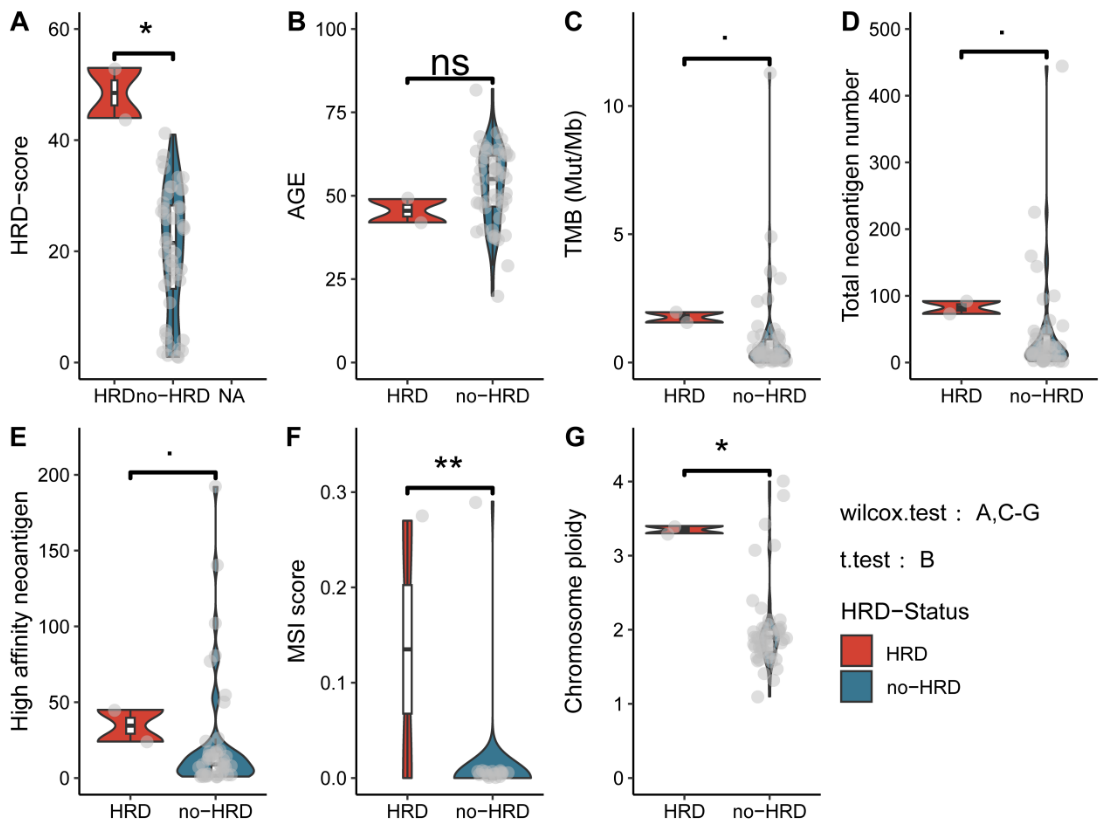

Figure S4. Differences in HRD score, TMB, TNB, and other indicators among different HRD statuses in the Hong Kong cohort.

The boxplots illustrate the variations in each indicator across different HRD statuses in the Hong Kong cohort, specifically (A) HRD score, (B) patient age, (C) tumour mutational burden (Mut/Mb), (D) total neoantigen number, (E) high affinity neoantigen, (F) MSI score, and (G) chromosome ploidy.

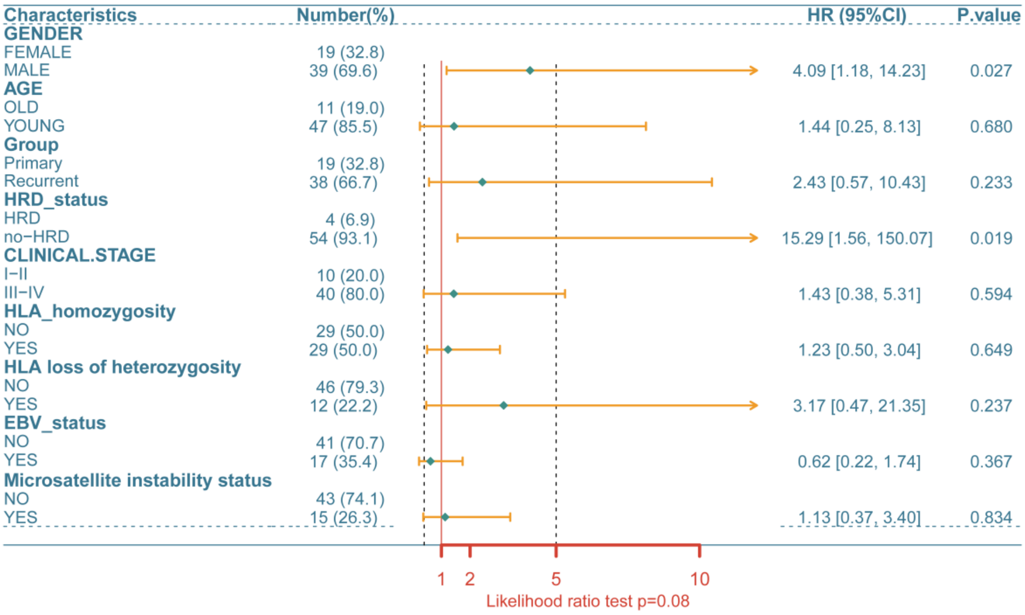

Figure S5. Results of multivariate Cox proportional survival regression analysis in the total population of the Zhujiang cohort.

Abbrevations: HRD = Homologous Recombination Deficiency; no−HRD = without Homologous Recombination Deficiency; EBV = Epstein-barr virus; HLA = Human leukocyte antigen. Symbols: OLD = 60 or more for age ; YOUNG = less than 60 for age

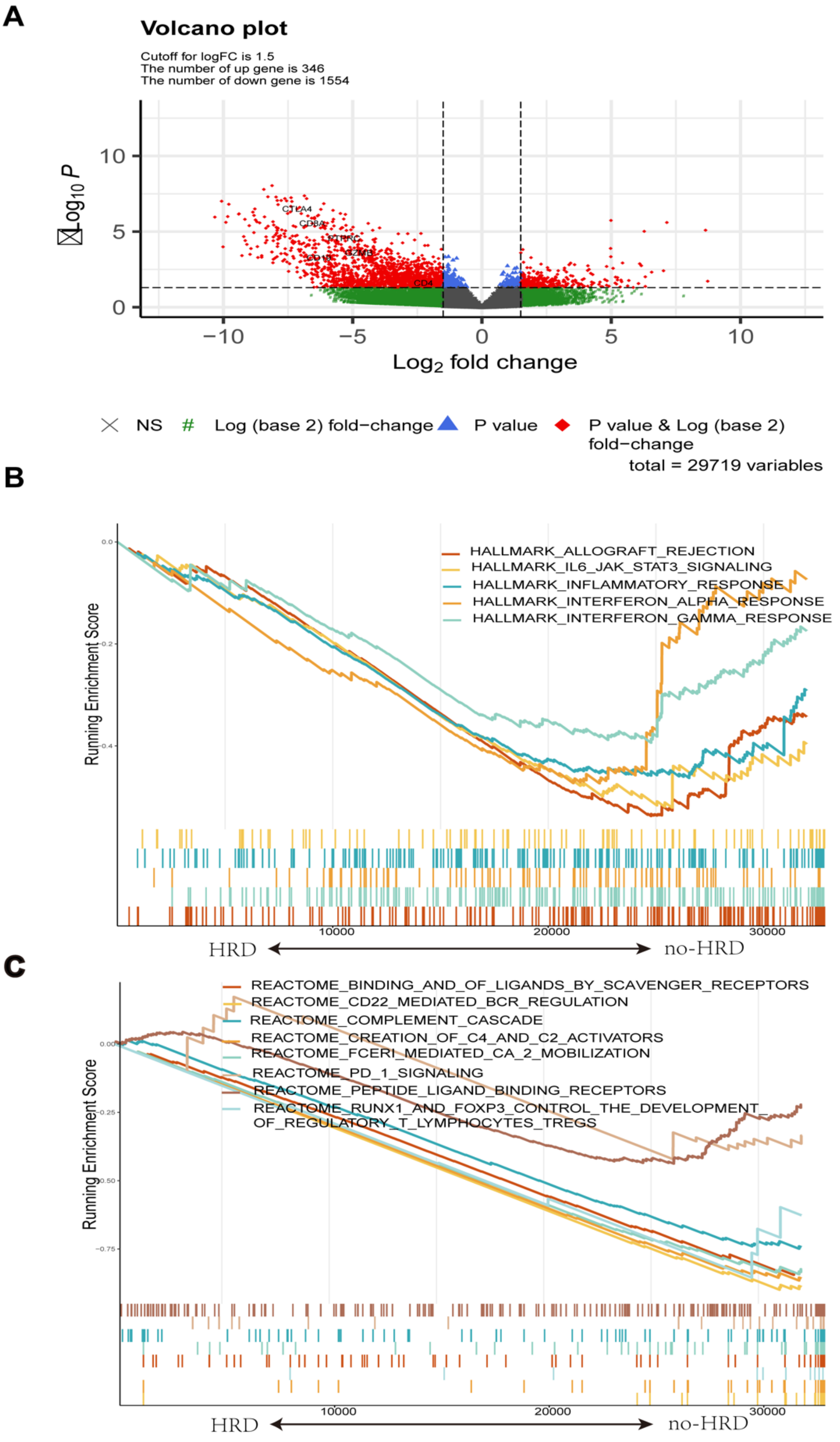

Figure S6. Plots of differential analysis and results of GSEA.

(A) Volcano plot of differential analysis between HRD and no-HRD group. (B) Ten immune-related HALLMARK pathways with $p < 0.05$ enriched in the no-HRD group and (C) eight immune-related REACTOME pathways with $p < 0.05$ enriched in the no-HRD group.

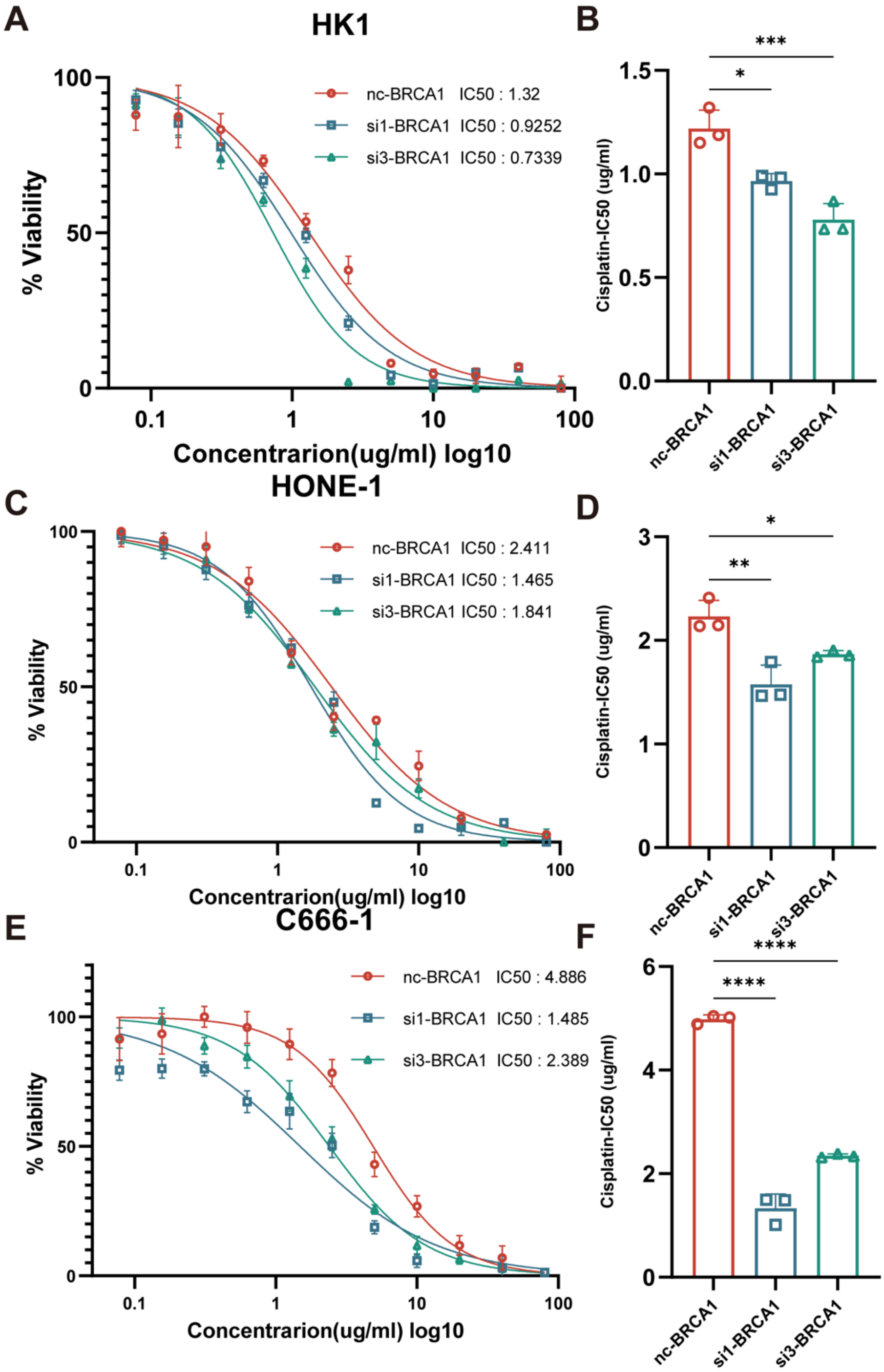

Figure S7. Knockdown of BRCA1 makes NPC cells more sensitive to chemotherapy (cisplatin).

(A,C,E). Dose-response curves of nc-BRCA1 group and si-BRCA1 groups to cisplatin in HK1, HONE-1 and C666-1 cell lines, respectively. (B,D,F). Changes in cisplatin IC50 after BRCA1 knockdown are presented as histograms for the three cell lines, respectively. The experiment was independently repeated three times.

(Student's t test, Wilcoxon rank-sum test, ****, $P < 0.0001$; ***, $P < 0.001$; **, $P < 0.01$; *, $P < 0.05$)
