# Supplementary material for: Homologous recombination deficiency (HRD) is associated with better prognosis and possibly causes a non‐inflamed tumour microenvironment in nasopharyngeal carcinoma
Source: J Pathol Clin Res. 2024 Aug 5;10(5):e12391. doi: 10.1002/2056-4538.12391 (PMC11300531; doi:10.1002/2056-4538.12391)
Supplement: Supplementary file 5 — Table S2. Clinical characteristics of the Zhujiang cohort according to HRD status Table S3. Clinical characteristics of the Singapore cohort according to HRD status Table S4. Clinical characteristics of the Hong Kong cohort according to HRD status [file CJP2-10-e12391-s002.pdf]

Homologous recombination deficiency (HRD) is associated with better prognosis and possibly causes a non-inflamed tumour microenvironment in nasopharyngeal carcinoma

X Zhou *et al*. *J Pathol Clin Res* https://doi.org/10.1002/2056-4538.12391

Supplementary Tables S2–S4

Table S2. Clinical characteristics of the Zhujiang Cohort according to HRD status (HRD versus no-HRD).

	HRD N=4	no-HRD N=54	p overall
GENDER:			0.577
FEMALE	2 (50.0%)	15 (28.8%)	
MALE	2 (50.0%)	37 (71.2%)	
AGE	32.2 (8.54)	43.7 (13.6)	0.066
AGE_binary:			1.000
OLD	0 (0.00%)	8 (15.7%)	
YOUNG	4 (100%)	43 (84.3%)	
Group:			1.000
Primary	1 (25.0%)	18 (34.0%)	
Relapse	3 (75.0%)	35 (66.0%)	
HLA-I homogeneity:			0.611
NO	1 (25.0%)	28 (51.9%)	
YES	3 (75.0%)	26 (48.1%)	
MSI_status:			0.052
NO	1 (25.0%)	41 (77.4%)	
YES	3 (75.0%)	12 (22.6%)	
CLINICAL.STAGE_binary:			0.571
I-II	0 (0.00%)	10 (21.7%)	
III-IV	4 (100%)	36 (78.3%)	
HLA-I LOH:			0.564
NO	4 (100%)	38 (76.0%)	
YES	0 (0.00%)	12 (24.0%)	
EBV_status:			0.283
NO	1 (33.3%)	30 (66.7%)	
YES	2 (66.7%)	15 (33.3%)	
APOBEC_Enriched:			1.000
no	3 (75.0%)	44 (81.5%)	
yes	1 (25.0%)	10 (18.5%)	

Table S3. Clinical characteristics of the Singapore cohort according to HRD status (HRD versus no-HRD).

	HRD N=12	no-HRD N=44	p overall
GENDER:			0.686
FEMALE	3 (25.0%)	8 (18.2%)	
MALE	9 (75.0%)	36 (81.8%)	
AGE	51.0 (13.2)	53.7 (7.90)	0.510
AGE_binary:			1.000
OLD	3 (25.0%)	10 (22.7%)	
YOUNG	9 (75.0%)	34 (77.3%)	
HLA-I homogeneity:			0.309
NO	10 (83.3%)	29 (65.9%)	
YES	2 (16.7%)	15 (34.1%)	
MSI_status:			1.000
NO	11 (91.7%)	38 (86.4%)	
YES	1 (8.33%)	6 (13.6%)	
CLINICAL.STAGE_binary:			0.588
I/II	1 (8.33%)	9 (20.5%)	
III/IV	11 (91.7%)	32 (72.7%)	
NO RECORD	0 (0.00%)	3 (6.82%)	
HLA-I LOH:			0.007
NO	5 (45.5%)	35 (87.5%)	
YES	6 (54.5%)	5 (12.5%)	
EBV.CNV_binary:			1.000
HIGH	6 (50.0%)	21 (50.0%)	
LOW	6 (50.0%)	21 (50.0%)	
APOBEC_Enriched:			1.000
no	10 (83.3%)	37 (86.0%)	
yes	2 (16.7%)	6 (14.0%)	
SMOKING.HISTORY:			0.286
NO	9 (75.0%)	22 (52.4%)	
YES	3 (25.0%)	20 (47.6%)	

Table S4. Clinical characteristics of the Hong Kong cohort according to HRD status (HRD versus no-HRD).

	HRD N=2	no-HRD N=48	p overall
GENDER:			0.363
FEMALE	1 (50.0%)	9 (18.8%)	
MALE	1 (50.0%)	39 (81.2%)	
AGE	45.5 (4.95)	53.0 (11.9)	0.230
AGE_binary:			0.542
OLD	0 (0.00%)	17 (35.4%)	
YOUNG	2 (100%)	31 (64.6%)	
STAGE:			1.000
I	0 (0.00%)	6 (12.5%)	
II	0 (0.00%)	9 (18.8%)	
III	1 (50.0%)	20 (41.7%)	
IV	1 (50.0%)	13 (27.1%)	
MSI_status:			0.079
NO	1 (50.0%)	47 (97.9%)	
YES	1 (50.0%)	1 (2.08%)	
HLA-I homogeneity:			1.000
NO	2 (100%)	36 (75.0%)	
YES	0 (0.00%)	12 (25.0%)	
HLA-I LOH:			0.343
NO	1 (50.0%)	38 (82.6%)	
YES	1 (50.0%)	8 (17.4%)	
